# CPEB3 inhibits epithelial-mesenchymal transition by disrupting the crosstalk between colorectal cancer cells and tumor-associated macrophages via IL-6R/STAT3 signaling

**DOI:** 10.1186/s13046-020-01637-4

**Published:** 2020-07-11

**Authors:** Qian Zhong, Yuxin Fang, Qiuhua Lai, Shanci Wang, Chengcheng He, Aimin Li, Side Liu, Qun Yan

**Affiliations:** Guangdong Provincial Key Laboratory of Gastroenterology, Department of Gastroenterology, Nanfang Hospital, Southern Medical University, 1838th North Guangzhou Avenue, Guangzhou, 510515 China

**Keywords:** Colorectal cancer, CPEB3, Cytoplasmic polyadenylation element binding protein 3, Tumor-associated macrophage, IL-6, EMT

## Abstract

**Background:**

Crosstalk between cancer cells and tumor-associated macrophages (TAMs) mediates tumor progression in colorectal cancer (CRC). Cytoplasmic polyadenylation element binding protein 3 (CPEB3) has been shown to exhibit tumor-suppressive role in CRC.

**Methods:**

The expression of CPEB3, CD68, CD86 and CD163 was determined in CRC tissues. SW480 or HCT116 cells overexpressing CPEB3 and LoVo or RKO cells with CPEB3 knockdown were constructed. Stably transfected CRC cells were co-cultured with THP-1 macrophages to determine the malignant phenotype of CRC cells, macrophage polarization, and secretory signals. The inhibition of CPEB3 on tumor progression and M2-like TAM polarization was confirmed in nude mice.

**Results:**

Decreased CPEB3 expression in CRC was associated with fewer CD86^+^ TAMs and more CD163^+^ TAMs. CPEB3 knockdown in CRC cells increased the number of CD163^+^ TAMs and the expression of IL1RA, IL-6, IL-4 and IL-10 in TAM supernatants. TAMs enhanced CRC cell proliferation and invasion via IL-6, and then activated the IL-6R/STAT3 pathway in CRC cells. However, CPEB3 reduced the IL-6R protein levels by directly binding to IL-6R mRNA, leading to decreased phosphorylated-STAT3 expression in CRC cells. CCL2 was significantly increased in CPEB3 knockdown cells, while CCL2 antibody treatment rescued the effect of CPEB3 knockdown in promoting CD163^+^ TAM polarization. Eventually, we confirmed that CPEB3 inhibits tumor progression and M2-like TAM polarization in vivo.

**Conclusions:**

CPEB3 is involved in the crosstalk between CRC cells and TAMs by targeting IL-6R/STAT3 signaling.

## Background

Colorectal cancer (CRC) is the third most common cancer and the second most common cause of cancer-associated deaths worldwide [1]. CRC development and progression are complex processes that are caused by a combination of accumulated genetic modifications in cancer cells and the surrounding microenvironment [2, 3]. CRC cells recruit vasculature and stroma (including immune cells, fibroblasts, cytokines, and the extracellular matrix that surrounds them) to the tumor microenvironment (TME); an activated TME, in turn, modifies the malignant behaviors of cancer cells [4]. Tumor-associated macrophages (TAMs) are one of the most abundant cell types in the TME, directly affecting tumor progression in many cases [5].

TAMs display two main phenotypes (M1 and M2), which usually have contrasting effects on tumor progression [6]. M1 macrophages, which are classically activated macrophages, are polarized by lipopolysaccharide (LPS) and interferon-γ (IFN-γ) [7]. M2 macrophages, the alternatively activated macrophages, are polarized in the presence of IL-4, IL-10, or IL-13 [8]. M1 macrophages with a high expression of CD86 are involved in the inflammatory response, pathogen clearance, and antitumor immunity [9, 10]. In contrast, M2 macrophages with a high expression of CD163 or CD206 play a key role in the anti-inflammatory response, wound healing, and pro-tumorigenic properties [11, 12]. TAMs closely resemble the M2-polarized macrophages, and high levels of M2 macrophage infiltration are associated with poor prognosis in colon cancer patients [13, 14]. Although several studies have reported that TAMs exhibit an anti-inflammatory phenotype, in recent years, activated TAMs have been shown to produce multiple pro-inflammatory cytokines, such as IL-6 [15]. IL-6 is involved in the induction of genes important for tumor cell cycle progression and apoptosis suppression [16]. It has been proven to play an important role in the immune regulation, the inflammatory response, and the epithelial-mesenchymal transition (EMT) of tumor cells [17, 18]. Notably, TAM-derived IL-6 binds to receptor/glycoprotein 130 (gp130) and upregulates Janus kinase (JAK)/STAT3 signaling in CRC cells, leading to increased EMT and chemoresistance [19, 20]. On the contrary, tumor cell-derived paracrine signals contribute to M2-like macrophages, including IL-10, CSF-1, different chemokines (CCL2, CCL18, CCL17, and CXCL4), and various extracellular matrix components [15, 21–23]. Therefore, the crosstalk between tumor cells and TAMs is a key step during tumor progression.

The cytoplasmic polyadenylation element (CPE) sequence was originally identified in mRNAs from *Xenopus* oocytes and was shown to bind a CPE-binding protein CPEB [24]. CPEB3, which is one of four different CPEB variants known today [25], binds the CPE sequence (UUUUUAU) in the 3′ untranslated regions of target mRNAs. CPEB3 is related to tumorigenesis and has been found to be downregulated in colorectal cancer through the microarray-based high-throughput screening [26]. The IncRNA SUMO1P3 epigenetically repressed the expression of CPEB3, and promoted cell proliferative ability and inhibited apoptotic ability in CRC [27]. Our previous research showed that CRC tissues exhibited decreased CPEB3 expression, a phenomenon that predicts poor prognosis for patients with CRC (unpublished data). However, the molecular mechanisms and regulatory network of CPEB3 in CRC are still unclear.

In this study, we investigated the role of CPEB3 in inhibiting TAM-induced EMT in CRC cells. Additionally, knockdown of CPEB3 promoted the secretion of CCL2 in CRC cells, promoting M2-like TAM polarization. Further mechanistic studies revealed that CPEB3 in CRC cells decreased the protein expression of IL-6R by directly binding to the 3’UTR of IL-6R mRNA, thus inhibiting the IL-6R/STAT3 signal transduction pathway. The results presented in here show that decreased CPEB3 expression results in CCL2-induced M2-like TAM polarization and IL-6-induced EMT in CRC cells, contributing to new insights concerning crosstalk between TME and CRC cells.

## Materials and methods

### Clinical samples

Human colorectal cancer and adjacent non-tumorous tissue samples for qRT-PCR analysis were obtained from a total of 82 patients who underwent surgical resection in the Department of General Surgery of Nanfang Hospital affiliated to Southern Medical University. Twenty colorectal cancer samples were randomly selected for immunohistochemistry (IHC) detection and analysis. All the samples were gathered with informed consent according to the Institutional Review Board of Ethical Committee–approved protocol.

### Cell culture and treatment

The human monocyte cell line THP-1 and CRC cell lines (SW480, HCT116, LoVo, and RKO) were obtained from the Shanghai Institute of Biochemistry and Cell Biology (Shanghai, China). Lentiviruses carrying full-length CPEB3 or short hairpin RNA (shRNA_CPEB3) sequences targeting against human CPEB3 mRNA and matched negative controls were constructed by the Shanghai Institute of Biochemistry and Cell Biology. SW480, HCT116, LoVo and RKO cells were transfected with the indicated lentivirus overnight, then 2 μg/mL puromycin was added after 72 h of transfection to obtain stably transfected CRC cells. For macrophage generation, THP-1 cells were treated with 100 ng/mL phorbol- 12-myristate-13-acetate (PAM) (Beyotime, Shanghai, China) for 12 h to differentiate into adhered macrophages. To obtain TAM supernatants, CRC cells were seeded in 0.4-μm pore inserts, then transferred to a 6-well plate seeded with THP-1 macrophages in advance and co-cultured for another 24 h. For co-culture experiments, stably transfected CRC cells were co-cultured with THP-1 macrophages for another 24 h.

### Animal models

Five-week-old BALB/c male mice were purchased from the Experimental Animal Center of Southern Medical University (Guangzhou, China) and sheltered under specific pathogen-free conditions. For tumor formation in mice, mice were randomly assigned to four groups (five mice per group): HCT116-CPEB3 group, LoVo-shCPEB3 group, and matched negative control groups. HCT116-Ctrl/CPEB3 (5 × 10^6^) and LoVo-shCtrl/shCPEB3 (5 × 10^6^) were subcutaneously injected into the right back portion of male BALB/c mice at five weeks of age. Tumor nodules were examined every five days and the volume was evaluated using the following formula: tumor volume = (width^2^ × length)/2. Mice were sacrificed after a period of 30 days and examined for the growth of subcutaneous tumors. For liver metastasis assay, LoVo-shCtrl/shCPEB3 (5 × 10^6^) were injected into the spleen of nude mice, then 5 mg/kg IL-6R inhibitor (tocilizumab) was injected intraperitoneally weekly. After 30 days, mice injected with CRC cells were sacrificed and livers were removed for examination. All animal care and handling procedures were performed in accordance with the NIH’s Guide for the Care and Use of Laboratory Animals.

### Cell proliferation and colony formation assay

Stably transfected CRC cells were treated with supernatants from TAMs (co-cultured with CRC cells), IL-6 (20 ng/mL, Peprotech, Rocky Hill, NJ, USA), tocilizumab (5 μg/mL, Selleck Chemicals, Houston, TX, USA) and neutralizing antibodies to IL-6 (anti-IL-6; R&D Systems, Minneapolis, MN, USA). The proliferation rate of these stably transfected CRC cells was assessed at 24, 48, 72 and 96 h using the Cell Counting Kit-8 (CCK-8; Beyotime). Each time-point was assessed in replicates of three wells. For the colony formation assay, the stable cell lines (400 cells/well) were seeded in 6 well plates. After 2 weeks, the cells were fixed in 4% paraformaldehyde and stained with crystal violet for 30 min at room temperature. Colonies consisting of > 50 cells were counted.

### Matrigel invasion assay

The Matrigel invasion assays were carried out in 24 well plates with 8 μm polycarbonate nucleopore filters (Corning, Tewksbury, MA, USA). The membrane for the invasion assay was covered with 100 μL BD Matrigel (diluted 1:4 with serum-free medium) in advance. The stable cells lines treated with or without tocilizumab (5 μg/mL, Selleck Chemicals) were seeded in the upper chambers; the lower chambers were filled with medium containing 10% FBS along with or without 20 ng/mL IL-6. In addition, the lower chambers were also filled with TAM cells supernatants along with or without neutralizing antibodies to IL-6 (anti-IL-6; R&D Systems). After a 48 h incubation, the cells adhering to the lower filter surface were counted.

### Flow cytometry

THP-1 macrophages were co-cultured with stably transfected CRC cells along with or without neutralizing antibodies to CCL2 (anti-CCL2; R&D Systems). Then these macrophages were processed into single cell suspensions, incubated with antibodies (BV421 Mouse anti-Human CD68, BB515 Mouse anti-Human CD86, PE Mouse anti-Human CD163, all from BD (BD Biosciences, San Jose, CA, USA) for 1 h at 4 °C. Mouse macrophages were then stained with CD206-APC (mouse), CD86-FITC (mouse), F4/80-PE (mouse) antibodies (eBiosciences, San Diego, CA, USA), respectively. Macrophages of nude mice subcutaneous tumor were separated and obtained using Percoll (Sigma-Aldrich, St. Louis, MO, USA) following the instruction. Flow cytometry was performed using a FACS Calibur flow cytometer (BD Biosciences). Flow cytometric analysis was performed on FlowJo software (FlowJo, Ashland, OR, USA).

### Immunohistochemistry

IHC staining was performed on 5-μm sections of paraffin-embedded tissue samples to detect the protein expression levels of CD68, CD86, CD163, CPEB3, E-cadherin, vimentin and Ki67. In brief, the slides were incubated in anti-CD68(1:500, Servicebio, Wuhan, China), anti-CD86 (1:100, BOSTER, Wuhan, China), anti-CD163 (1:500, BOSTER), anti-CPEB3 (1:200, Abcam, Cambridge, MA, USA), anti-E-cadherin (1:1000, Proteintech, Chicago, IL, USA), vimentin (1:1000, Proteintech) and Ki67 (1:1000, Proteintech) antibodies at 4 °C overnight. All slides were independently evaluated by two observers. The score for CPEB3, E-cadherin and vimentin staining was based on the integrated staining intensity and the proportion of positive cells. IHC staining of CD68, CD86, CD163 and Ki67 was calculated by the positive cell numbers in the per high field. All the percentages/numbers of positive cells were expressed as the average of six randomly selected microscopic fields.

### Western blot analysis

Protein extracts were probed with antibodies against human CPEB3 (1:500, Abcam), phospho-STAT3(Tyr705) (1:1000, Cell Signaling Technology, Danvers, MA, USA), STAT3 (1:1000, Cell Signaling Technology), IL-6R (1:500, Santa Cruz Biotechnology, Santa Cruz, CA, USA), IL-6ST (1:500, Santa Cruz Biotechnology), phospho-JAK1(Y1022/1023) (1:1000, ABclonal Technology, Wuhan, China), JAK1 (1:1000, ABclonal Technology), ZEB2 (1:1000, Proteintech), E-cadherin (1:1000, Proteintech), N-cadherin (1:1000, Proteintech), vimentin (1:1000, Proteintech), slug (1:1000, Proteintech), snail1 (1:1000, Proteintech), and GAPDH (1:1000, Proteintech). Peroxidase-conjugated anti-mouse (1:5000, Proteintech) or rabbit antibody (1:2000, Proteintech) was used as a secondary antibody and the antigen-antibody reaction was visualized by an enhanced chemiluminescence assay (Millipore, Bedford, MA, USA).

### Luminex assays

The levels of cytokines in cell culture supernatants were measured using IFN-γ, IL-1β, IL-1RA, IL-4, IL-6, IL-10, IL-12p70, IL23, IP10, TNF-α and CCL2 Human ProCartaPlex™ simplex kit and Human Basic kit (eBioscience). Briefly, 50 μL samples or standard recombinant protein dilution were added to a mixture of capture beads coated with related monoclonal antibodies to a group of cytokines, washed beads were further incubated with biotin-labeled anti-human cytokine antibodies for 1 h at room temperature followed by incubation with streptavidin–phycoerythrin for 30 min. Samples were analyzed using Luminex 200™ (Luminex Corporation, Austin, TX, USA).

### Enzyme-linked immunosorbent assay (ELISA)

The cytokine of IL-6 in cells supernatants was estimated by ELISA, using a commercial kit (MultiSciences, Hangzhou, China), according to the manufacturer’s instructions. Positive controls were supplied in the kit.

### RNA isolation, reverse transcription (RT) and real-time PCR

Total RNA from tissues and cultured cell lines was isolated using the Trizol reagent (TaKaRa, Osaka, Japan) according to the manufacturer’s instruction, and cDNA was synthesized using random primers and the TaKaRa PrimeScript RT regent kit. qRT-PCR reactions were performed in triplicate using the SYBR Green method on a Light Cycler 480 Real Time PCR System (Roche Diagnostics, Mannheim, Germany). The PCR primers are listed in Supplementary Table S1.

### RNA immunoprecipitation (RIP)

RNA immunoprecipitation of CPEB3 targeting RNAs was performed in SW480 and LoVo cells. Briefly, SW480 and LoVo cells were lysed in a polysome lysis buffer according to the Magna RIP RNA-Binding Protein Immunoprecipitation Kit guidelines (Millipore). RNA was extracted for RT-PCR and qRT-PCR detection.

### Luciferase reporter assay

SW480-Ctrl, SW480-CPEB3, HCT116-Ctrl and HCT116-CPEB3 cells of 80% confluence were transfected with indicated plasmids using Lipofectamine 3000 (Invitrogen, Carlsbad, CA, USA). Wild-type and mutated forms of the IL6R-3′ UTR were subcloned into a pmir-GLO vector were co-transfected per well of a 24-well plate. Cell extracts were prepared at 36 h after transfection. The luciferase activity was measured with a Dual Luciferase Reporter Assay System (Promega, Madison, WI, USA).

### Statistical analysis

All statistical analyses were performed using Graph-Pad Prism software (version 6.0, GraphPad Software, San Diego, CA, USA). Pearson’s correlation analysis was performed to assess the relationship between CD86, CD163 expression and CPEB3 expression in patients with CRC. Groups of discrete variables were compared using the Student’s t test or nonparametric ANOVA. All experiments for cell cultures were performed independently at least three times and in triplicates each time. In all in vitro experiments, data represented at least three independent experiments and are expressed as means ± SEM. In in vivo experiments, data are expressed as the mean ± SEM. *P*-values < 0.05 were considered statistically significant (in all figures: *, *p* < 0.05; **, *p* < 0.01; ***, *p* < 0.001; ****, *p* < 0.0001; ns = not significant).

## Results

Decreased CPEB3 in human CRC correlates with low CD86^+^ TAM content and high CD163^+^ TAM content.

**Fig. 1:**
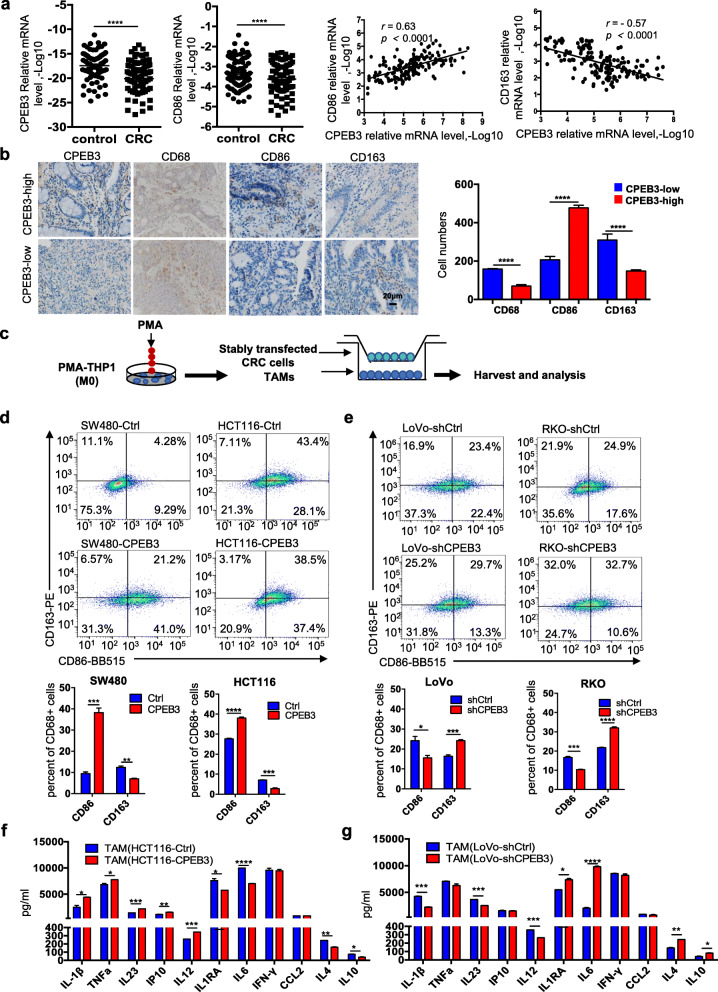
Decreased CPEB3 in human CRC correlates with low CD86^+^ TAM content and high CD163^+^ TAM content **(a)** The expression of CPEB3 and CD86 in 82 pairs of CRC tissues and adjacent non-tumor tissues was detected using qRT-PCR. Correlation between CPEB3 and CD86 or CD163 expression levels in 82 colorectal cancer tissues; error bars, SEM. **(b)** The protein expression of CPEB3, CD68, CD86, and CD163 in a human colorectal cancer tissue array was detected by IHC staining. Representative photos are shown (400× magnification). The number of CD68^+^, CD86^+^ and CD163^+^ cells per high-power field in tissues from colorectal cancer patients with different levels of CPEB3 expression; error bars, SEM. **(c)** Schema for an in vitro model of stably transfected CRC cells co-cultured with TAMs. **(d)** Flow cytometry was used to explore the surface expression of CD86 and CD163 in SW480-Ctrl/CPEB3 and HCT116-Ctrl/CPEB3 cells; error bars, SEM. **(e)** Flow cytometry was used to explore the surface expression of CD86 and CD163 in LoVo-shCtrl/shCPEB3 and RKO-shCtrl/shCPEB3 cells; error bars, SEM. **(f)** We measured the expression of the respective inflammatory cytokines in cell culture supernatants of TAMs co-cultured HCT116-Ctrl/CPEB3 cells using ProcartaPlex combinable panels; error bars, SEM. **(g)** We measured the expression of the respective inflammatory cytokines in cell culture supernatants of TAM-co-cultured LoVo-shCtrl/shCPEB3 cells using ProcartaPlex combinable panels; error bars, SEM; ns, not significant; * *P* < 0.05; ** *P* < 0.01; *** *P* < 0.001; **** *P* < 0.0001

To evaluate how CPEB3 in CRC cells may inhibit M2-like TAM polarization, we first assessed the expression of CPEB3, CD86 and CD163 in 82 pairs of CRC tissues and adjacent non-cancer tissues using qRT-PCR. CPEB3 and CD86 mRNA expression was decreased in CRC tissues compared with controls (Fig. 1a). The patients’ information of the samples was provided in Supplement Fig. 1a. Correlation analysis showed that CPEB3 mRNA expression was positively correlated with CD86 mRNA (*r* = 0.63, *p* < 0.0001) and negatively correlated with CD163 mRNA (*r* = − 0.57, *p* < 0.0001) (Fig. 1a). We also examined the expression of CPEB3 protein and macrophage infiltration in CRC tissues from 20 patients using IHC. The number of CD86^+^ TAMs was significantly lower in tumor tissues with low CPEB3 expression than that in tumor tissues with high CPEB3 expression, but the number of CD163^+^ TAMs was significantly increased in tumor tissues with low CPEB3 expression than that in tumor tissues with high CPEB3 expression (Fig. 1b). Furthermore, we utilized an in vitro model of stably transfected CRC cells co-cultured with TAMs (Fig. 1c). CRC cells with stably overexpressing CPEB3 or shRNA-CPEB3 were generated (Supplementary Fig. 1b – d). The overexpression of CPEB3 in SW480 (SW480-CPEB3) and HCT116 (HCT116-CPEB3) cells decreased the number of CD163^+^ TAMs differentiated from THP-1 macrophages (Fig. 1d). Knockdown of CPEB3 in LoVo (LoVo-shCPEB3) and RKO cells (RKO-shCPEB3) significantly increased the number of CD163^+^ TAMs (Fig. 1e). Taking the Fig. 1d as an example, we have placed the results of the flow-cytometry gating strategy in Supplement Fig. 1e, the same as the gating strategy of other flow-cytometry used in this study. Interestingly, flow cytometry identified the CD86 and CD163 double-positive TAMs after co-culture with stably transfected CRC cells, indicating that tumor cells induced TAMs of a mixed M1/M2 phenotype. The cytokines of a typical M1 phenotype (IL-1β, TNF-α, IL23, IP10, and IL-12p70) and M2 phenotype (IL-1RA, IL-6, IFN-γ, CCL2, IL-4, and IL-10) were investigated using Luminex assays. THP-1 macrophages co-cultured with HCT1116-CPEB3 cells showed increased IL-1β, TNF-α, IL23, IP10, and IL-12p70, and significantly decreased IL1RA, IL-6, IL-4, and IL-10, illustrating a predominant M1 phenotype (Fig. 1f). In contrast, THP-1 macrophages co-cultured with LoVo-shCPEB3 cells showed increased IL1RA, IL-6, IL-4, and IL-10, and decreased IL-1β, IL23, and IL-12p7, indicating a predominant M2 phenotype (Fig. 1g). These results supported the hypothesis that CPEB3 expression in CRC cells inhibits macrophage differentiation into the M2-like phenotype in the CRC cell milieu.

### CPEB3 inhibits the TAM-induced EMT in CRC cells

**Fig. 2:**
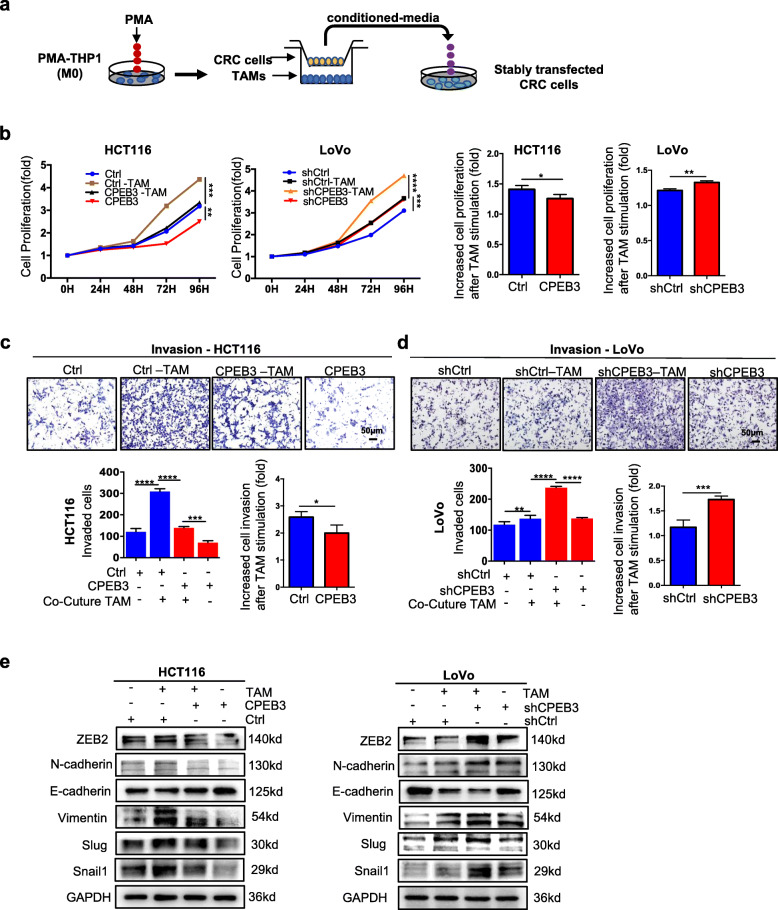
CPEB3 inhibits the TAM-induced EMT in CRC cells **(a)** Schema for stably transfected CRC cells treated with conditioned media from TAMs. **(b)** Cell Counting Kit-8 was used to quantify the number of HCT116-Ctrl/CPEB3 and LoVo-shCtrl/shCPEB3 cells, which were then cultured with supernatants from TAMs; error bars, SEM. **(c)** The invasion of HCT116-Ctrl/CPEB3 cells (co-cultured TAMs) was measured by a Transwell assay (200× magnification); error bars, SEM. **(d)** The invasion of LoVo-shCtrl/shCPEB3 cells (co-cultured TAMs) was measured by a Transwell assay (200× magnification); error bars, SEM. **(e)** The effect of the TAMs on the invasion of CRC cells (HCT116-Ctrl/CPEB3 and LoVo-shCtrl/shCPEB3) was analyzed by western blot analysis; error bars, SEM. * *P* < 0.05; ** *P* < 0.01; *** *P* < 0.001; **** *P* < 0.0001

TAM has been shown to promote the EMT in tumor cells [19]. To investigate whether CPEB3 plays a role in the regulation of TAM-induced EMT in CRC cells, we treated the HCT116-CPEB3 and LoVo-shCPEB3 cells with TAM supernatants (Fig. 2a). The cell proliferation assay showed that the TAM supernatants significantly promoted the cell proliferation, while the ratio of increased cell proliferation after TAM stimulation was significantly reduced in the HCT116-CPEB3 group compared to the HCT116-Ctrl group (Fig. 2b). The ratio of increased cell proliferation after TAM stimulation was significantly higher in the LoVo-shCPEB3 group than that in the LoVo-shCtrl group (LoVo-shCtrl vs. LoVo-shCtrl, *P* < 0.05). Similarly, Matrigel invasion was strongly promoted by the TAM supernatants (Fig. 2c – d). The ratio of increased invasion was significantly lower in the HCT116-CPEB3 group (HCT116-Ctrl vs HCT116-CPEB3, *P* < 0.05) (Fig. 2c), while it was higher in the LoVo-shCPEB3 group (LoVo-shCtrl vs. LoVo-shCPEB3, *P* < 0.05) (Fig. 2d). Western blot data further showed that the expression of epithelial marker E-cadherin was decreased, while the mesenchymal marker ZEB2, N-cadherin, vimentin, slug and snail1 were increased in the CRC cells treated with TAM supernatants (Fig. 2e and Supplementary Fig. 2a – d). Taken together, these results showed that the expression of CPEB3 inhibited the TAM-induced EMT in CRC cells.

### CPEB3 inhibits the EMT induced by TAM-derived IL-6 in CRC cells

**Fig. 3:**
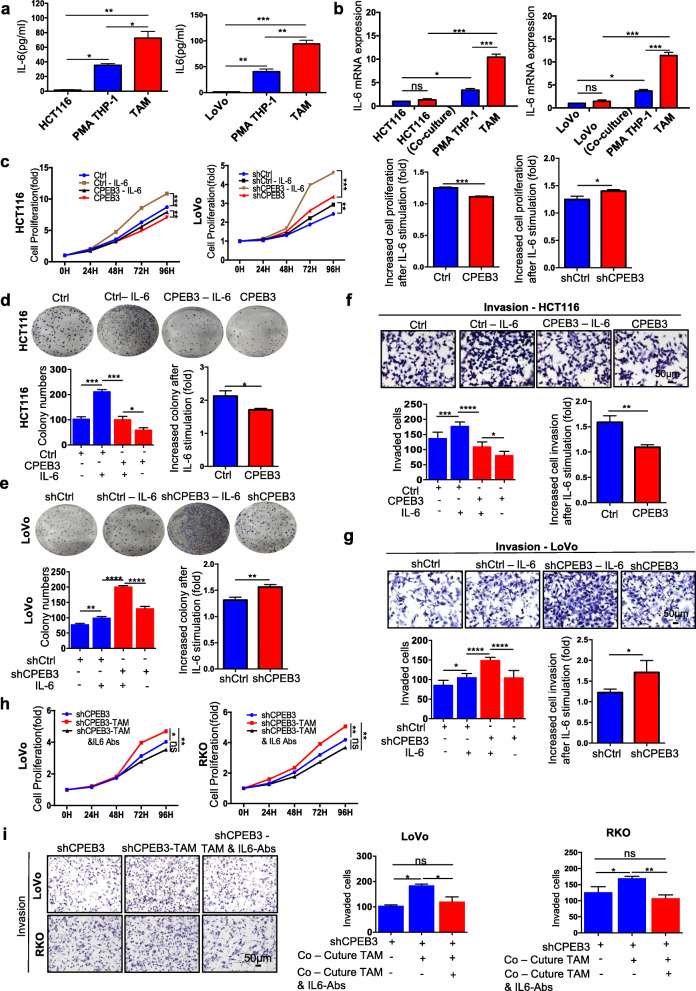
CPEB3 inhibits EMT induced by TAM-derived IL-6 in CRC cells **(a)** IL-6 expression was detected in THP-1 macrophages, HCT116, or LoVo, and TAMs with 24 h of co-culture using ELISA; error bars, SEM. **(b)** IL-6 expression was detected in HCT116 or LoVo and TAMs with or without 24 h of co-culture using qRT-PCR; error bars, SEM. **(c)** Cell Counting Kit-8 was used to quantify the number of HCT116-Ctrl/CPEB or LoVo-shCtrl/shCPEB3 with or without IL-6 (20 ng/mL); error bars, SEM. **(d)** A colony formation assay was used to quantify the number of spheres of IL-6 (20 ng/mL)-supplemented HCT116-CPEB3 cells and the control; error bars, SEM. **(e)** A colony formation assay was used to quantify the number of spheres of IL-6 (20 ng/mL)-supplemented LoVo-shCPEB3 cells and the control; error bars, SEM. **(f)** The invasion of IL-6 (20 ng/mL)-supplemented HCT116-CPEB3 cells and the control was measured by a Transwell assay (200× magnification); error bars, SEM. **(g)** The invasion of IL-6 (20 ng/mL)-supplemented LoVo-shCPEB3 cells and the control was measured by a Transwell assay (200× magnification); error bars, SEM. **(h)** Cell Counting Kit-8 was used to quantify the cell numbers of LoVo/RKO-shCPEB3 cells, cultured TAM supernatants, and IL-6-depleted TAM supernatants. **(i)** The invasion of LoVo/RKO-shCPEB3 cells, co-cultured TAMs, and IL-6-depleted TAMs co-cultured with LoVo or RKO cells was measured by a Transwell assay (200× magnification); error bars, SEM. * *P* < 0.05; ** *P* < 0.01; *** *P* < 0.001; **** *P* < 0.0001

Given that cytokine secretion represents the major functional response of macrophages, it was speculated that a signaling mechanism between TAMs and CRC cells exists that accounts for at least part of the previously described pro-tumorigenic activities [28, 29]. To identify the TAM-derived factors, we conducted a qRT-PCR analysis of 11 cytokines related to the inflammation/EMT axis. The levels of IL-6 mRNA were significantly upregulated and abundant in the supernatants of TAMs co-cultured with LoVo-shCPEB3 cells, while they were significantly reduced in the supernatants of TAMs co-cultured with HCT116-CPEB3 cells (Supplementary Fig. 3a – b). ELISA further showed that IL-6 levels were significantly increased in the media from TAMs co-cultured with HCT116 cells compared to those from THP-1 macrophages or HCT116 cells alone (Fig. 3a). In TAMs co-cultured with LoVo cells, similar results were obtained (Fig. 3a). To confirm that increased secretion of IL-6 was derived from TAMs and not from CRC cells, we detected IL-6 mRNA in HCT116 or LoVo cells, and found that the level of IL-6 mRNA was low and showed no difference after co-culturing with THP-1 macrophages (Fig. 3b). In addition, the levels of IL-6 mRNA were significantly increased in TAMs than in THP-1 macrophages, and HCT116 or LoVo cells co-cultured with THP-1 macrophages promoted IL-6 expression in TAMs but not in HCT116 or THP-1 macrophages (Fig. 3b). These results suggested that most of the IL-6 was derived from TAMs, consistent with the ELISA results (Fig. 3a).

To evaluate whether IL-6 was critical for the role of TAM-mediated EMT in CRC cells, an exogenous recombinant IL-6 was added to the culture medium of the stably transfected CRC cells. The results showed that IL-6 significantly promoted the cell proliferation (Fig. 3c), colony formation (Fig. 3d – e) and invasive abilities (Fig. 3f – g) of the HCT116-Ctrl/CPEB3 and LoVo- shCtrl/shCPEB3 groups. The ratio of increased cell proliferation, colony formation and Matrigel invasion after IL-6 stimulation was lower in the HCT116-CPEB3 group than that in the HCT116-Ctrl group (HCT116-Ctrl vs. HCT116-CPEB3, *P* < 0.05), while it was higher in the LoVo-shCPEB3 group than in the LoVo-shCtrl group (LoVo-shCtrl vs. LoVo-shCPEB3, *P* < 0.05) (Fig. 3c – g). Similar results were found in the SW480-CPEB3 and RKO-shCPEB3 groups (Supplementary Fig. 4a – e). Furthermore, supplementation with IL-6 neutralizing antibody in the conditioned media from TAMs inhibited the TAM-induced proliferation ability and invasive ability of LoVo/RKO-shCPEB3 (Fig. 3h – i). Therefore, co-culture with CRC cells induced THP-1 macrophages to become TAMs with increased IL-6 mRNA production and IL-6 secretion, which is critical for TAM-mediated EMT in CRC cells.

### CPEB3 inhibits IL-6R/STAT3 signaling via direct binding to IL-6R mRNA in CRC cells

**Fig. 4:**
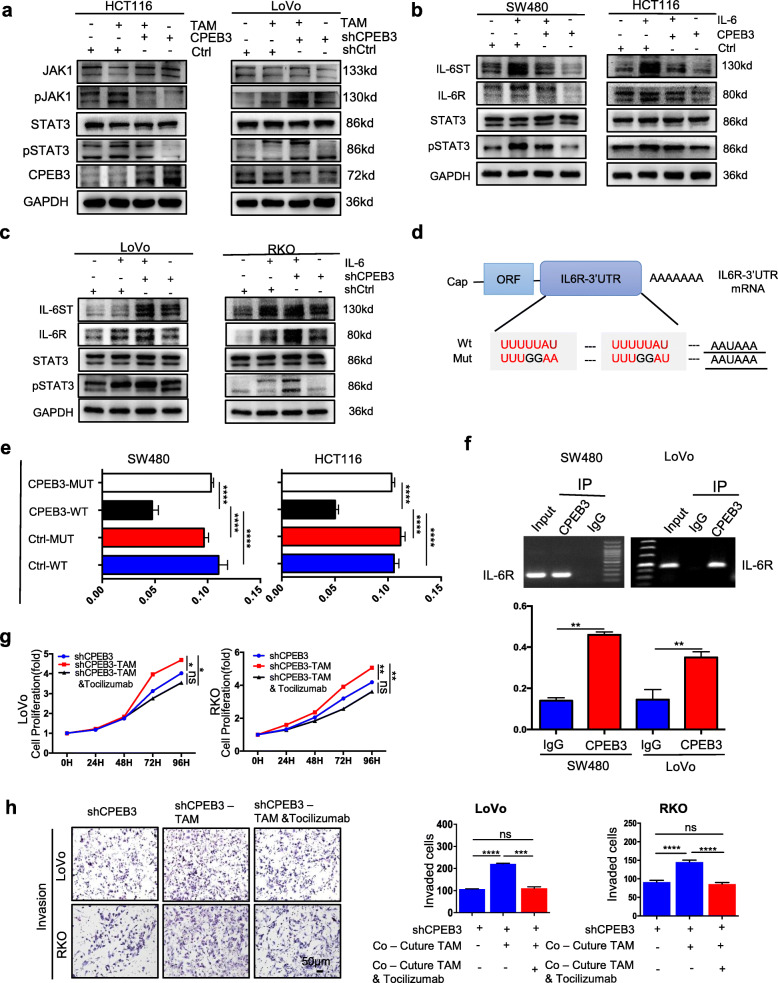
CPEB3 inhibits IL-6R/STAT3 signaling via direct binding to IL-6R mRNA in CRC cells **(a)** The effect of the TAMs on the JAK1, pJAK1, pSTAT3, and STAT3 in CRC cells (HCT116-Ctrl/CPEB3 and LoVo-shCtrl/shCPEB3) was analyzed by western blot analysis. **(b)** The effect of IL-6 on the IL-6ST, IL-6R, STAT3, and pSTAT3 in CRC cells (SW480-Ctrl/CPEB3 and HCT116-Ctrl/CPEB3) was analyzed by western blot analysis. **(c)** The effect of IL-6 on the IL-6ST, IL-6R, STAT3, and pSTAT3 in CRC cells (LoVo-shCtrl/shCPEB3 and RKO-shCtrl/shCPEB3) was analyzed by western blot analysis. **(d)** Schematic diagram of IL-6R-3′ -UTR reporter mRNA. **(e)** Luciferase assays were performed to detect the binding activity of CPEB3 and IL-6R. Relative fold-change in luciferase activity is shown; error bars, SEM. **(f)** RT-PCR of the RIP products confirmed the direct binding capacity of CPEB3 to the IL-6R-3′ -UTR in SW480 and LoVo cells. qRT-PCR of the RIP products further confirmed the direct binding capacity of CPEB3 to the IL-6-3′-UTR in SW480 and LoVo cells. Input, 5% of total lysate; error bars, SEM. **(g)** Cell Counting Kit-8 was used to quantify the number of LoVo/RKO-shCPEB3 cells, cultured TAMs supernatants, and TAMs supernatants treated with tocilizumab (5 ng/mL). **(h)** The invasion of LoVo/RKO-shCPEB3 and LoVo/RKO-shCPEB3 co-cultured TAMs treated with or without tocilizumab (5 ng/mL) was measured by a Transwell assay (200× magnification); error bars, SEM. ns, not significant; * *P* < 0.05; ** *P* < 0.01; *** *P* < 0.001; **** *P* < 0.0001

Our previous genomics results showed that CPEB3 mainly regulates the IL-6R/STAT3 pathway, which is one of the major recognized effector pathways of IL-6 (unpublished data). We next aimed to determine whether CPEB3 inhibits IL-6R/STAT3 signaling in CRC cells. Western blot analysis showed that TAM induced the expression of pJAK1 and pSTAT3 in the HCT116-Ctrl/CPEB3 and LoVo-shCtrl/shCPEB3 groups (Fig. 4a). The ratio of increased pJAK1/JAK1 and pSTAT3/STAT3 expression after TAM stimulation was significantly lower in the HCT116-CPEB3 group than in the HCT116-Ctrl group (HCT116-Ctrl vs. HCT116-CPEB3, *P* < 0.05), while it was higher in the LoVo-shCPEB3 group than in the LoVo-shCtrl group (LoVo-shCtrl vs. LoVo-shCPEB3, *P* < 0.05) (Supplementary Fig. 5a – b). IL-6 promoted the expression of IL-6ST, IL-6R, STAT3, and pSTAT3 in the SW480-Ctrl/CPEB3, HCT116-Ctrl/CPEB3 groups (Fig. 4b), LoVo-shCtrl/shCPEB3, and RKO-shCtrl/shCPEB3 (Fig. 4c). The average gray values of IL-6ST, IL-6R, STAT3, and pSTAT3 were analyzed in the SW480-Ctrl/CPEB3, HCT116-Ctrl/CPEB3 groups (Supplementary Fig. 6a – b) and LoVo-shCtrl/shCPEB3, RKO-shCtrl/shCPEB3 (Supplementary Fig. 7a – b). Similarly, the ratio of increased IL-6ST, IL-6R, and pSTAT3/STAT3 expressions after IL-6 stimulation were significantly lower in the SW480-CPEB3 and HCT116-CPEB3 groups than in the control group (Supplementary Fig. 6a – b), while they were higher in the LoVo-shCPEB3 and RKO-shCPEB3 groups than that in control group (Supplementary Fig. 7a – b). However, the mRNA expression of IL-6ST and IL-6R in stably transfected CRC cells was not different between the groups (Supplementary Fig. 8a – b). In silico analyses of the IL-6R mRNA sequence showed that the 3′ UTR of IL-6R mRNA contains two potential CPEB3-binding cytoplasmic polyadenylation elements (CPEs; with a consensus sequence of UUUUUAU) and one U-rich sequence in addition to the polyadenylation signal (AAUAAA), which is responsible for CPEB3-mediated mRNA 3′ UTR regulation (Fig. 4d). The reporter assays showed that overexpression of CPEB3 markedly inhibited the luciferase activity in CRC cells transfected with the 3′ UTR sequence of IL-6R containing wild-type CPEs, but this repression effect was abrogated in CRC cells transfected with mutant CPEs (Fig. 4e). Furthermore, endogenous CPEB3 binds IL-6R mRNA directly in SW480 and LoVo cells (Fig. 4f). Subsequently, we found that tocilizumab, a humanized monoclonal antibody that binds to IL-6R, blocked the proliferative ability of LoVo/RKO-shCPEB3 cells promoted by TAM supernatants (Fig. 4g). Meanwhile, treatment with tocilizumab also inhibited TAM-induced invasive abilities of LoVo/RKO-shCPEB3 cells (Fig. 4h). These data suggest that CPEB3 in CRC cells inhibits IL-6R/STAT3 signaling by directly binding to CPEs in the 3′ UTR of IL-6R mRNA.

### CPEB3 modulates CCL2 secretion in CRC cell supernatants to regulate TAM polarization

**Fig. 5:**
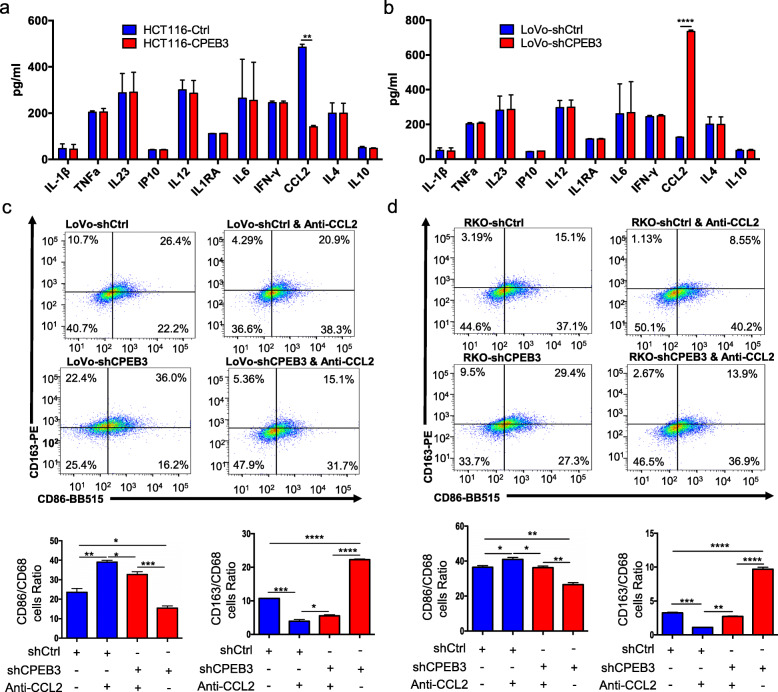
CPEB3 modulates CCL2 secretion in CRC cell supernatants to regulate TAM polarization **(a)** We measured the expression of the respective inflammatory cytokines in cell culture supernatants of HCT116-Ctrl/CPEB3 cells by ProcartaPlex combinable panels; error bars, SEM. **(b)** We measured the expression of the respective inflammatory cytokines in the supernatants of LoVo-shCtrl/shCPEB3 cells by ProcartaPlex combinable panels; error bars, SEM. **(c)** THP-1 macrophages were co-cultured with LoVo-shCtrl/shCPEB3 with or without CCL2-neutralizing antibody (1 μg/mL) for 24 h. Flow cytometry was used to explore the surface expression of CD86 and CD163 in the differentiated macrophages; error bars, SEM. **(d)** THP-1 macrophages were co-cultured with RKO-shCtrl/shCPEB3 cells with or without a CCL2-neutralizing antibody (1 μg/mL) for 24 h. Flow cytometry was used to explore the surface expression of CD86 and CD163 in the differentiated macrophages. Error bars, SEM. * *P* < 0.05; ** *P* < 0.01; *** *P* < 0.001; **** *P* < 0.0001

To determine how CPEB3 in CRC cells regulates TAM polarization via IL-6R/STAT3 signaling, we used Luminex assays to screen for major cytokines, which may induce TAM differentiation in the culture supernatants of stably transfected CRC cells, including IFN-γ, IL-1β, IL-1RA, IL-4, IL-6, IL-10, IL-12p70, IL23, IP10, TNF-α, and CCL2. The results indicated that CCL2 secretion was significantly reduced in HCT116-CPEB3 cell supernatants (Fig. 5a), but increased in LoVo-shCPEB3 cell supernatants (Fig. 5b). CCL2 has been proven to be targeted and regulated by STAT3 [30–32]. To confirm the role of CCL2 in TAM polarization, flow cytometry was performed to analyze the TAM markers in a co-culture system composed of LoVo-shCtrl/shCPEB3 or RKO-shCtrl/shCPEB3 cells, and TAMs. The results showed that the expression of CD163 was increased while CD86 was decreased in TAMs co-cultured with LoVo-shCPEB3 or RKO-shCPEB3 cells compared to TAMs co-cultured with LoVo-shCtrl or RKO-shCtrl cells, respectively (Fig. 5c – d). Supplementation with a CCL2-neutralizing antibody in the supernatant inhibited the expression of CD163 but promoted the expression of CD86 in TAMs (Fig. 5c – d). Taken together, CPEB3 decreased the secretion of CCL2 in CRC cells and induced TAM polarization to the M1-like phenotype.

### CPEB3 attenuates tumorigenesis and inhibits CD163^+^ TAM polarization in vivo

**Fig. 6:**
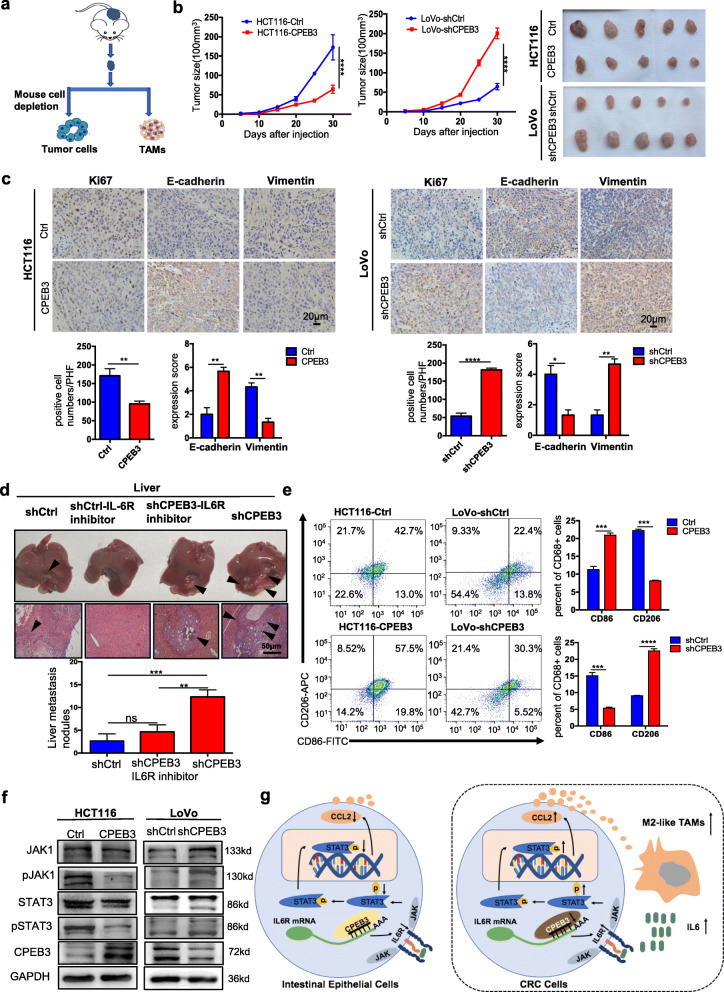
CPEB3 attenuates tumorigenesis and TAM polarization in vivo **(a)** Schematic of the procedure for separating tumor cells and TAMs. **(b)** HCT116 cells were stably infected with Ctrl and CPEB3 lentivirus, and LoVo cells were stably infected with shCtrl and shCPEB3 sequences. Tumorigenesis assay of Balb/c nude mice subcutaneously injected with HCT116-Ctrl/CPEB3 cells and LoVo-shCtrl/shCPEB3 cells (*n* = 20). Representative photos of tumors from mice in various groups. (**c)** IHC staining of Ki67 positive cells was counted per high-power field (PHF), while E-cadherin and vimentin expression scores were counted in tumor tissues in a mouse xenograft model; error bars, SEM. **(d)** The mice with intra-spleen injection of LoVo-shCtrl/shCPEB3 cells were treated with tocilizumab (5 mg/kg) weekly via intraperitoneally injection. The number of liver metastatic sites (indicated by arrows) was counted under the microscope; error bars, SEM. **(e)** Macrophages were separated from murine tumor tissues using Percoll-layered liquid. Surface expression of CD86 and CD163 was detected in macrophages using flow cytometry. The percentage of CD86^+^ or CD163^+^ cells in macrophages was reported using error bars and SEM. **(f)** Expression of JAK1, pJAK1, STAT3, and pSTAT3 in the tumor tissues of the two groups were analyzed by western blot analysis. **(g)** Schematic overview of the mechanisms by which CPEB3 modulate TAM polarization and inhibit colorectal cancer EMT. ** *P* < 0.01; *** *P* < 0.001; **** *P* < 0.0001

To explore the effect of CPEB3 on CRC cell EMT and macrophage polarization in vivo, a subcutaneous xenograft model of CPEB3-transduced CRC cells in BALB/c nude mice was constructed (Fig. 6a). As expected, we observed that the overexpression of CPEB3 led to the suppression of tumor growth compared to the control group, while knockdown of CPEB3 in LoVo cells promoted tumor growth (Fig. 6b). The number of Ki67-positive cells was lower in the HCT116-CPEB3 group than in the HCT116-Ctrl group. In addition, the expression of E-cadherin was increased, and the expression of vimentin was reduced in the HCT116-CPEB3 group than that in HCT116-Ctrl group, while contrary results were found in the LoVo-shCPEB3 group (Fig. 6c). In order to explore the expression of CPEB3 and IL-6 regulating the invasion of CRC cells, we constructed a liver metastasis model by injecting the LoVo-shCPEB3 CRC cells into the spleen of nude mice. Then, IL-6R inhibitor (5 mg/kg, tocilizumab) was intraperitoneally into nude mice weekly. As expected, there were more hepatic metastatic colonies in mice injected with LoVo-shCPEB3 cells than that injected with LoVo-shCtrl cells. Nevertheless, these effects were rescued after blockade of IL-6R inhibitor (tocilizumab). There were no hepatic metastatic colonies in mice injected with LoVo-shCtrl cells subsequently exposed to IL-6R inhibitor treatments (Fig. 6d). More CD86^+^ cells and less CD163^+^ cells were found in the HCT116-CPEB3 group than in the HCT116-Ctrl group. However, more CD163^+^ cells and less CD86^+^ cells were found in the LoVo-shCPEB3 group than in the LoVo-shCtrl group (Fig. 6e). Western blot analysis showed that the overexpression of CPEB3 in HCT116 cells inhibited pJAK1 and pSTAT3 in vivo, while the reverse was found in the LoVo-shCPEB3 group (Fig. 6f). The average gray values of pJAK1/JAK1, pSTAT3/STAT3, and CPEB3 were also analyzed in the above four groups (Supplementary Fig. 9a – b). In conclusion, we found that CPEB3 inhibits the EMT of CRC cells and CD163^+^ TAM polarization in vivo (Fig. 6g).

## Discussion

The key findings of our study include the demonstration that a decreased expression of CPEB3 in CRC cells is related to M2-like TAM polarization in human CRC tissues. We show that the knockdown of CPEB3 in CRC cells promotes CD163^+^ TAM polarization and M2-like TAM-derived cytokine production in a co-culture system. The secretory signals between stably transfected CRC cells and THP-1 macrophages were evaluated by Luminex assays, in which IL-6 from TAMs and CCL2 from CRC cells were determined. CPEB3 inhibits EMT induced by TAM-derived IL-6 in CRC cells. In addition, CPEB3 inhibits M2-like TAM polarization by regulating CCL2 secretion in CRC cells. Mechanistically, we show that CPEB3 in CRC cells inhibits IL-6R expression by directly binding to the 3′ UTR of IL-6R mRNA, contributing to an impaired IL-6 signal response and decreased downstream CCL2 secretion. Collectively, these results shed new light on the role of CPEB3 in the TME of CRC and provide a mechanistic basis for TAM polarization and TAM-induced EMT of CRC cells.

As an important component in TME, TAMs interact with tumor cells and plays a key role in tumor progression [33]. Tumor cell products (such as IL-10, IL-4, and CCL2) affect the M1/M2 transformation of TAMs, and TAMs, in turn, regulate the biological behavior of tumor cells by secreting small molecular substances [34]. As a pro-inflammatory cytokine, IL-6 has a marked effect on the microenvironments of a wide range of cancers [35], and the level of IL-6 could predict the progress and poor prognosis of colorectal cancer [20]. It is not a new concept that TAM-derived IL-6 promotes EMT of CRC cells [19], but we found that this effect was blocked with CPEB3 overexpression CRC cells. Our previous RNA-seq data revealed that CPEB3 significantly inhibits the IL-6R/STAT3 signal pathway in CRC cells, one of the most important pathways in response to IL-6 [36]. Data from our whole-genome expression arrays (accession number SE137306) are available from https://www.ncbi.nlm.nih.gov/geo/subs/. Christoph Becker et al. showed that colon cancer tissue showed significantly higher levels of IL-6R than normal colon tissue [37]. We found that IL-6R protein levels were decreased in overexpressed CPEB3 CRC cells, while IL-6R mRNA levels did not change. CPEB3 regulates target molecules at the post-transcriptional level, and our previous RIP-seq data suggested that IL-6R was enriched in the precipitates of CPEB3. In the present study, we confirmed the interaction of CPEB3 and IL-6R mRNA via RIP-PCR, and two potent CPEB3 binding sequences were found in the 3′ UTR of IL-6R mRNA. Tocilizumab, as an FDA-approved humanized monoclonal antibody against IL-6R, has been proposed to inhibit the trastuzumab-resistant HER2(+) breast cancer [38]. We found that the proliferation and invasion of CPEB3 knockdown CRC cells were inhibited when tocilizumab was added to the TAM supernatants. Therefore, we speculated that CPEB3 directly binds to the 3′ UTR of IL-6R mRNA, translationally regulates the expression of IL-6R protein, and further inhibits the IL-6/IL-6R/STAT3 signaling pathway in CRC cells.

We further discovered that CPEB3 regulates the M1/M2 transformation of TAMs, in addition to inhibiting the TAM-induced EMT of CRC cells. The following results support this conclusion. First, our results showed that the decreased expression of CPEB3 correlates with high levels of CD163 in human CRC tissues. Second, CD86^+^ cells were significantly increased, while CD163^+^ cells were significantly decreased in THP-1 macrophages co-cultured with overexpressed CPEB3 CRC cells, which showed significantly higher expression of IL-1β, TNF-α, IL23, IP10, and IL-12p70, illustrating a predominantly M1 phenotype. Contrasting results were observed in THP-1 macrophages co-cultured with knockdown CPEB3 CRC cells, which exhibited higher levels of the M2 markers IL1RA, IL-6, IL-4, and IL-10. We speculated that CPEB3 may regulate the expression of certain molecules in the CRC cell supernatants and further invert the polarization of TAMs. Therefore, we screened the changes in a panel of cytokines in stably transfected CPEB3 CRC cells, and CCL2 was identified as the only significantly upregulated cytokine in LoVo-shCPEB3 cells. A previous study reported that the IL-6/STAT3/FoxQ1 signal axis could promote macrophage infiltration through CCL2 in CRC [19]. Additionally, CCL2 from CRC cells could also foster vascularization and intravasation [39]. Tumor-derived CCL2 shapes macrophage polarization by GM-CSF and M-CSF [40], and positively correlates with TAM infiltration, tumor vascularization, and angiogenesis [41]. Along this line, CCL2 expression shifts human peripheral blood CD11b^+^ cells toward the M2-polarized phenotype [22]. In hepatocellular Carcinoma, FoxQ1 expression promotes macrophage infiltration through the VersicanV1/CCL2 axis [42]. In breast cancer, inflammatory monocytes can be continually recruited by CCL2 produced by cancer cells and differentiate into TAMs that facilitate the subsequent growth of metastatic cells [43, 44]. Consistent with previous results, knockdown of CPEB3 induced more CD163^+^ TAMs, whereas CCL2 blockade led to an enhanced expression of the M1-polarization-associated marker CD86 and diminished expression of M2-associated marker CD163 in TAMs. These data cumulatively showed that CPEB3 regulates the IL-6R/STAT3 signal axis to affect the secretion of downstream CCL2, which plays an imperative role in TAM polarization in CRC cell supernatants.

## Conclusion

In summary, CPEB3 inhibits IL-6R/STAT3 signaling by binding to IL-6R mRNA in CRC cells, regulating the crosstalk between TAMs and CRC cells. CPEB3 inhibits the upstream molecule TAM-derived IL-6, which promotes the proliferation and invasion of CRC cells. Meanwhile, CPEB3 inhibits the secretion of its downstream molecule CCL2 in CRC cells and inverts the polarization of M2-like TAMs.

## Supplementary information

**Additional file 1.**

## Data Availability

The datasets used and/or analyzed during the current study are available. from the corresponding author on reasonable request.

## Abbreviations

CRC: Colorectal cancer
CPEB3: Cytoplasmic polyadenylation element binding protein 3
CPE: Cytoplasmic polyadenylation element
TAM: Tumor-associated macrophages
TME: Tumor microenvironment
gp130: Glycoprotein 130
JAK: Janus kinase
LPS: Lipopolysaccharide
IFN-γ: Interferon-γ
EMT: Epithelial-mesenchymal transition
ELISA: Enzyme-linked immunosorbent assay
RIP: RNA immunoprecipitation
IHC: Immunohistochemistry
