# Supplementary material for: CPEB3 inhibits epithelial-mesenchymal transition by disrupting the crosstalk between colorectal cancer cells and tumor-associated macrophages via IL-6R/STAT3 signaling
Source: J Exp Clin Cancer Res. 2020 Jul 11;39:132. doi: 10.1186/s13046-020-01637-4 (PMC7353816; doi:10.1186/s13046-020-01637-4)
Supplement: Supplementary file 1 — Additional file 1. [file 13046_2020_1637_MOESM1_ESM.pdf]

Supplementary Information

a

Variable	n	Variable	n	Variable	n
**Age (years)**		**Gender**		**Pathological grade**	
< 70	58	Male	53	Well	51
≥ 70	24	Male	29	Moderate- Poor	31
**pT status**		**pN status**		**pM status**	
T1-2	11	N0	50	M0	69
T3-4	71	N1	32	M1	13
**Tumor stage**		**Tumor size**		**Tumor number**	
I/II	15	≤ 5 cm	46	Single	80
III/IV	67	> 5 cm	36	Multiple	2

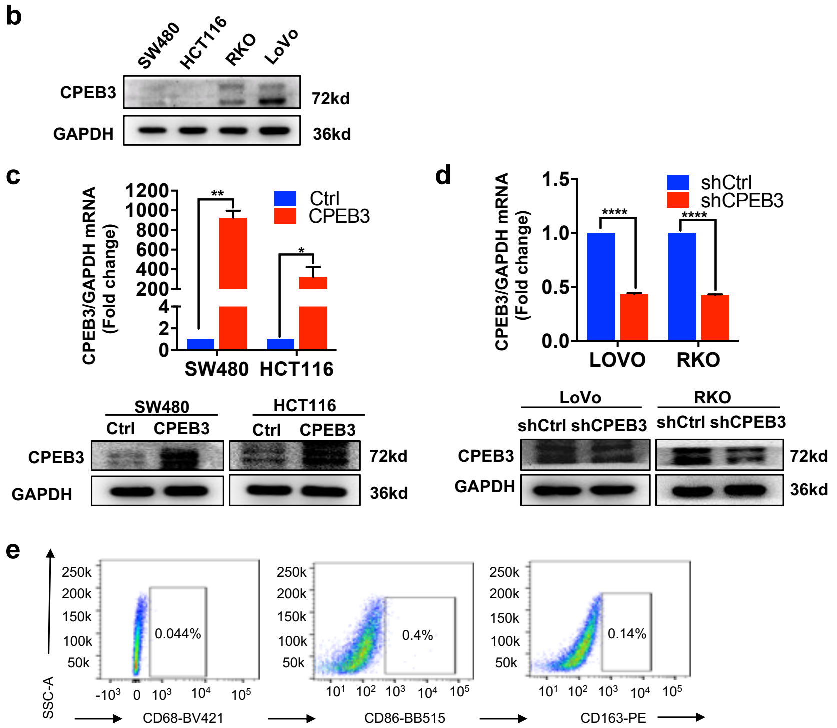

Supplementary Figure S1

CRC stable cell lines were constructed (a) The patients' information of the samples used in this study. (b) Expression of CPEB3 was detected by western blot in a panel of human colon cancer cell lines. (c) SW480 and HCT116 cells were stably transfected with Ctrl and CPEB3. The expression of CPEB3 in CPEB3 stably overexpressed and control SW480 and HCT116 cells was detected using qRT-PCR and western blot; error bars, SEM. (d) LoVo and RKO cells were stably transfected with shCtrl and shCPEB3. The expression of CPEB3 in CPEB3 stably depleted and control LoVo and RKO cells was detected using qRT-PCR and western blot. (e) The gating strategy of the flow-cytometry used in this study; error bars, SEM. *, $P < 0.05$; **, $P < 0.01$; ***, $P < 0.001$; ****, $P < 0.0001$.

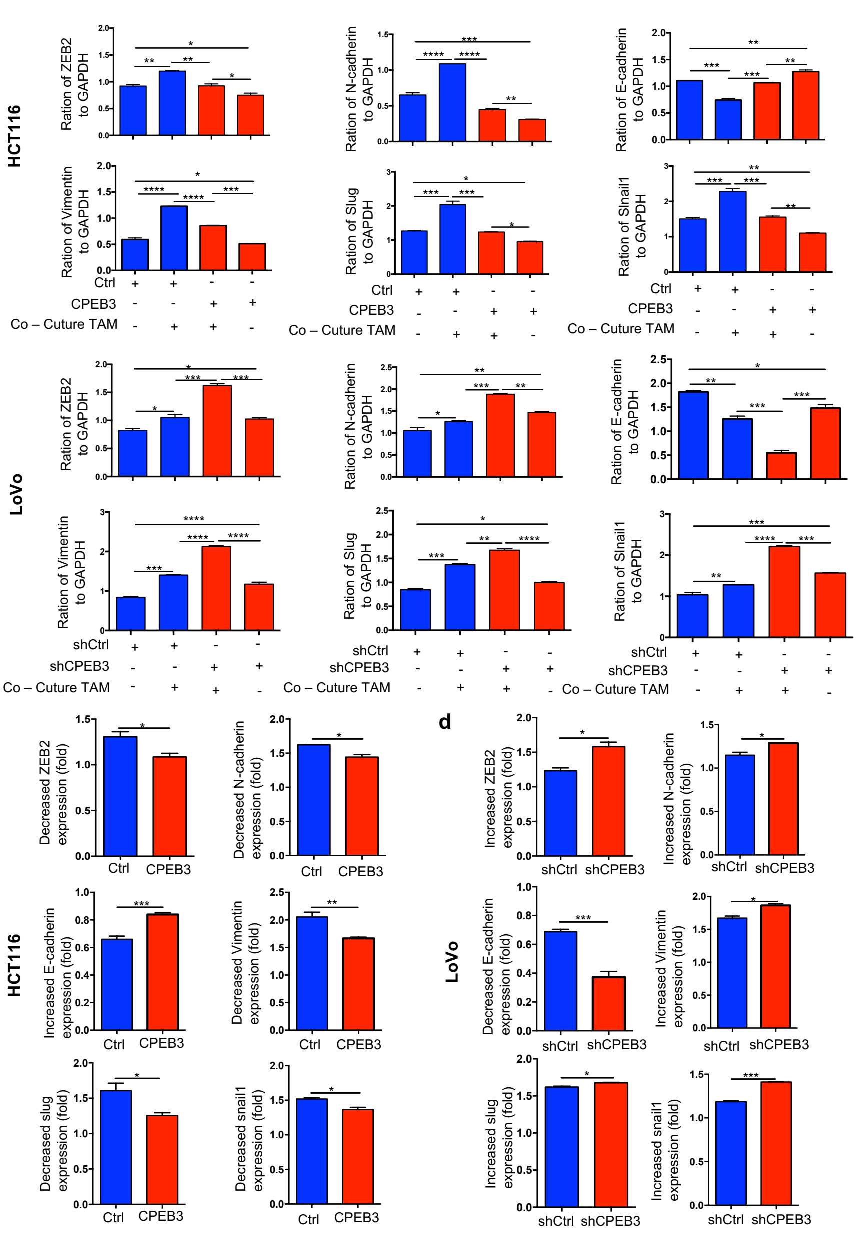

Supplementary Figure S2

The average gray values analysis of protein (a) The average gray values of ZEB2, N-Cadherin, E-Cadherin, vimentin, slug and snail1 in HCT116-Ctrl/CPEB3 cells were analyzed (as indicated in Fig. 2e); error bars, SEM. (b) The average gray values of ZEB2, N-Cadherin, E-Cadherin, vimentin, slug and snail1 in LoVo-shCtrl/shCPEB3 cells were analyzed (as indicated in Fig. 2e); error bars, SEM. (c)The ratio of increased protein expression was analyzed in Supplementary Fig. 2a; error bars, SEM. (d) The ratio of increased protein expression was analyzed in Supplementary Fig. 2b; error bars, SEM.*, $P < 0.05$; **, $P < 0.01$; ***, $P < 0.001$; ****, $P < 0.0001$.

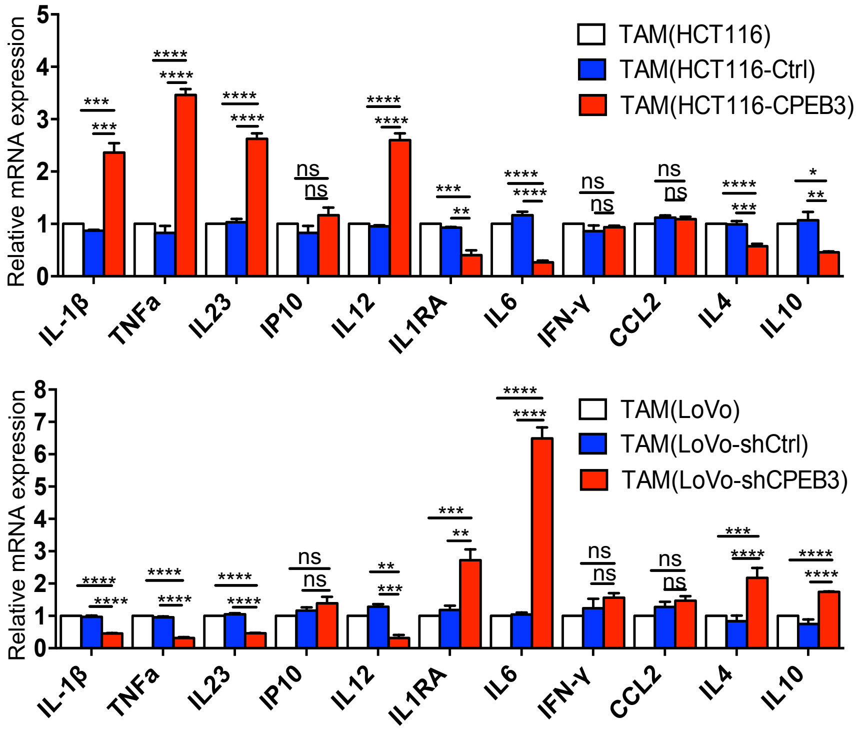

Supplementary Figure S3

Cytokine expression profiles in TAM in modulation of CPEB3 (a) Relative gene expression of M1 marker (IL1β, TNF-a, IL23, IP10 and IL12) and M2 marker (IL1RA, IL6, IFN-γ, CCL2, IL4 and IL10) were detected using qRT-PCR in the THP-1 macrophages cocultured with HCT116 and HCT116- Ctrl/CPEB3; error bars, SEM. (b) The above cytokines were detected using qRT-PCR in the THP-1 macrophages cocultured with LoVo and LoVo-shCtrl/shCPEB3 cells; error bars, SEM. *, $P < 0.05$; **, $P < 0.01$; ***, $P < 0.001$; ****, $P < 0.0001$.

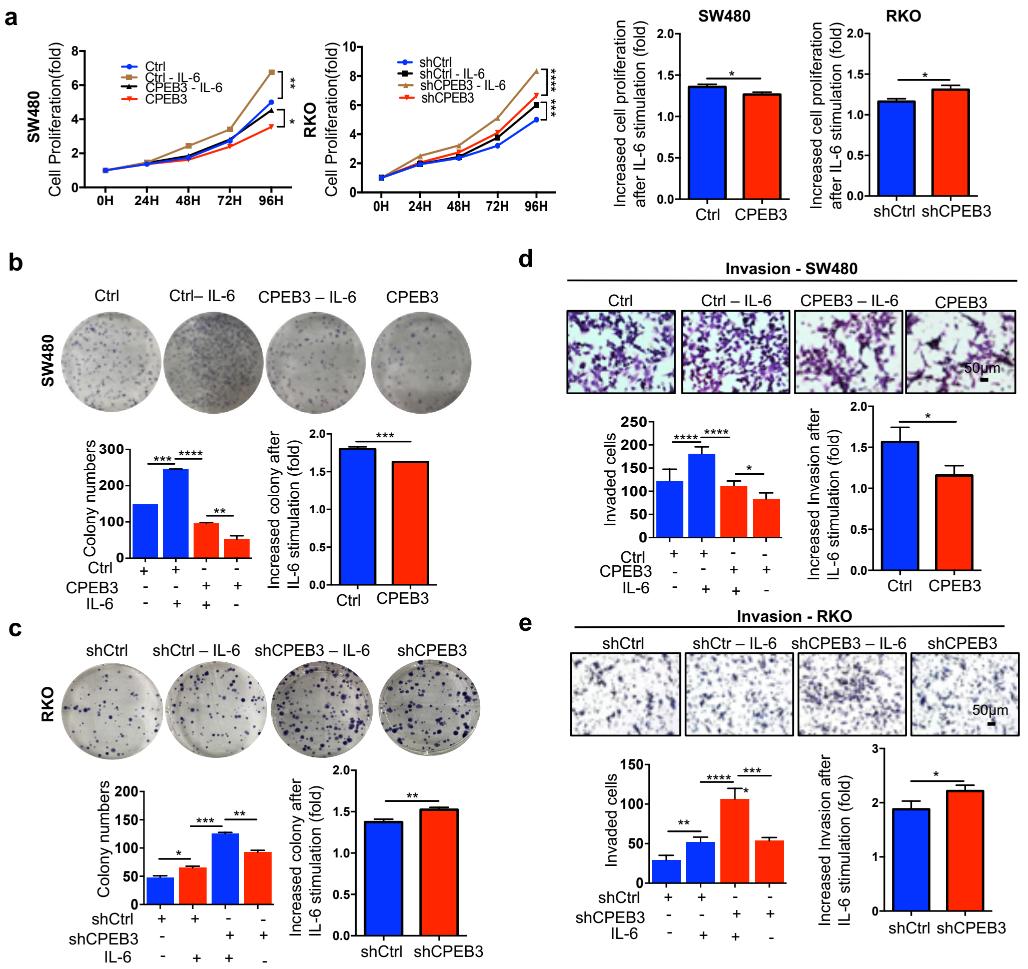

Supplementary Figure S4

IL-6 is required for TAMs-induced EMT of CRC cells (a) Cell Counting Kit-8 was used to quantify the number of SW480-Ctrl/CPEB3 or RKO-shCtrl/shCPEB3 cells with or without IL-6 (20ng/mL); error bars, SEM. (b) Colony formation assay was used to quantify the number of spheres of IL-6 (20ng/mL)-supplemented SW480-CPEB3 cells and its control; error bars, SEM. (c) Colony formation assay was used to quantify the number of spheres of IL-6 (20ng/mL)-supplemented RKO-shCPEB3 cells and its control; error bars, SEM. (d) The invasion of IL-6 (20ng/mL)-supplemented SW480-CPEB3 and its control was measured by a Transwell assay (200× magnification); error bars, SEM. (e) The invasion of IL-6 (20ng/mL)-supplemented RKO-shCPEB3 and its control was measured by a Transwell assay (200× magnification); error bars, SEM. *, $P < 0.05$; **, $P < 0.01$; ***, $P < 0.001$; ****, $P < 0.0001$.

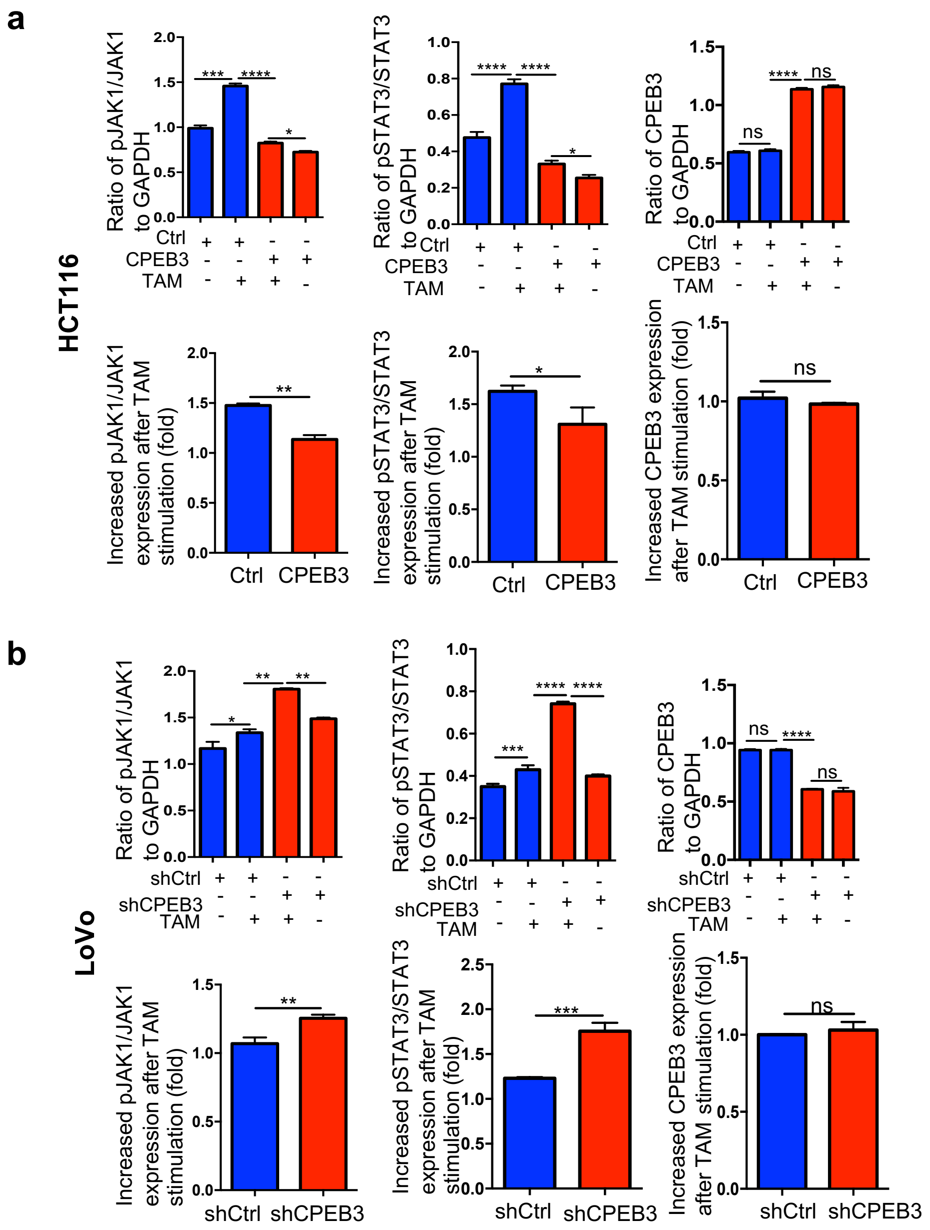

Supplementary Figure S5

The average gray values analysis of protein (a) The average gray values of pJAK1/JAK1, pSTAT3/STAT3 and CPEB3 in HCT116-Ctrl/CPEB3 cells were analyzed. The ratio of increased protein expression was analyzed (as indicated in Fig. 4a); error bars, SEM. (b) The average gray values of pJAK1/JAK1, pSTAT3/STAT3 and CPEB3 in LoVo-shCtrl/shCPEB3 cells were analyzed. The ratio of increased protein expression was analyzed (as indicated in Fig. 4a); error bars, SEM. *, $P < 0.05$; **, $P < 0.01$; ***, $P < 0.001$; ****, $P < 0.0001$.

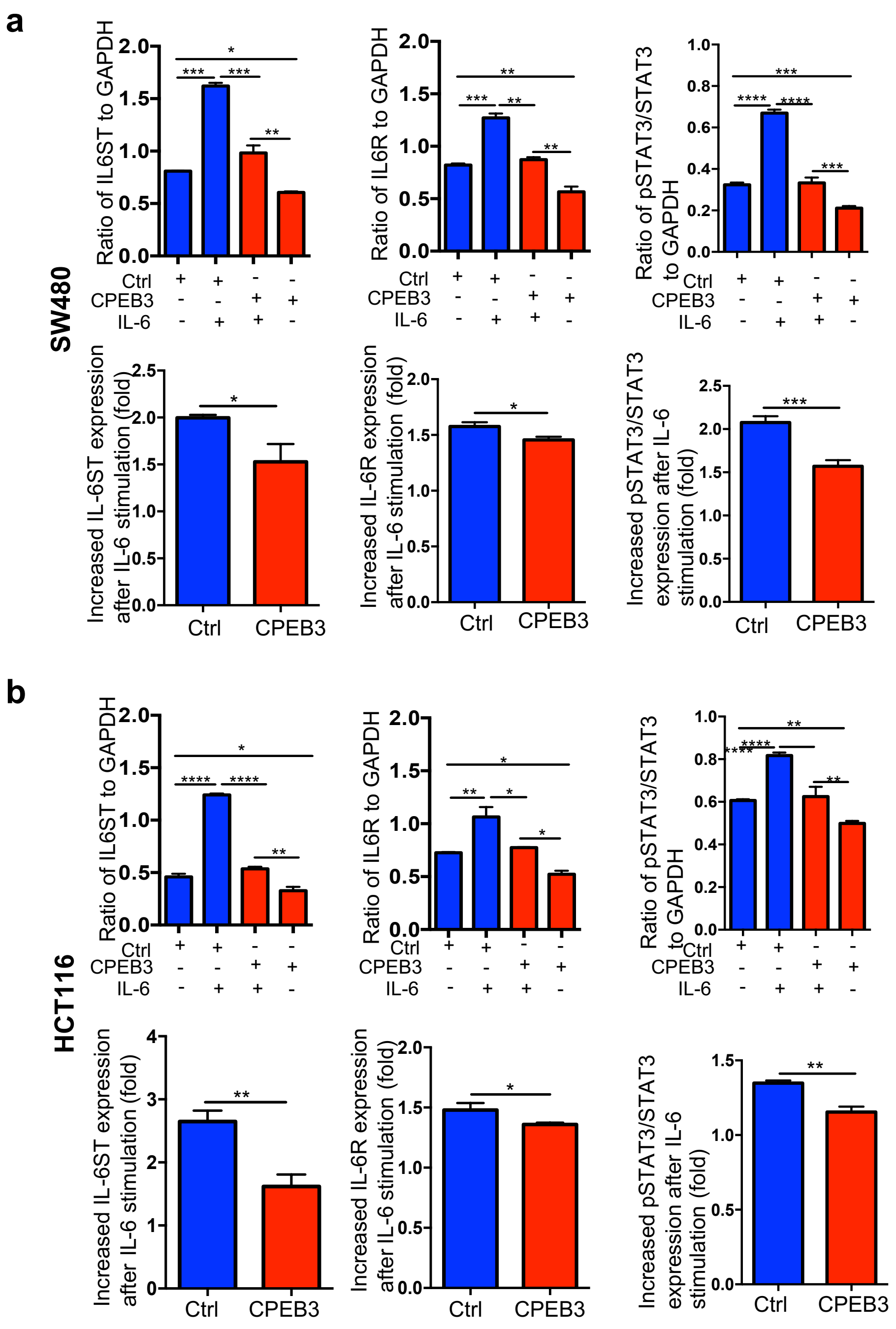

Supplementary Figure S6

The average gray values analysis of protein (a) The average gray values of IL-6ST, IL-6R and pSTAT3/ STAT3 in SW480-Ctrl/CPEB3 cells were analyzed. The ratio of increased protein expression was analyzed (as indicated in Fig. 4b); error bars, SEM. (b) The average gray values of IL-6ST, IL-6R and pSTAT3/STAT3 in HCT116-Ctrl/CPEB3 cells were analyzed. The ratio of increased protein expression was analyzed (as indicated in Fig. 4b); error bars, SEM. *, $P < 0.05$; **, $P < 0.01$; ***, $P < 0.001$; ****, $P < 0.0001$.

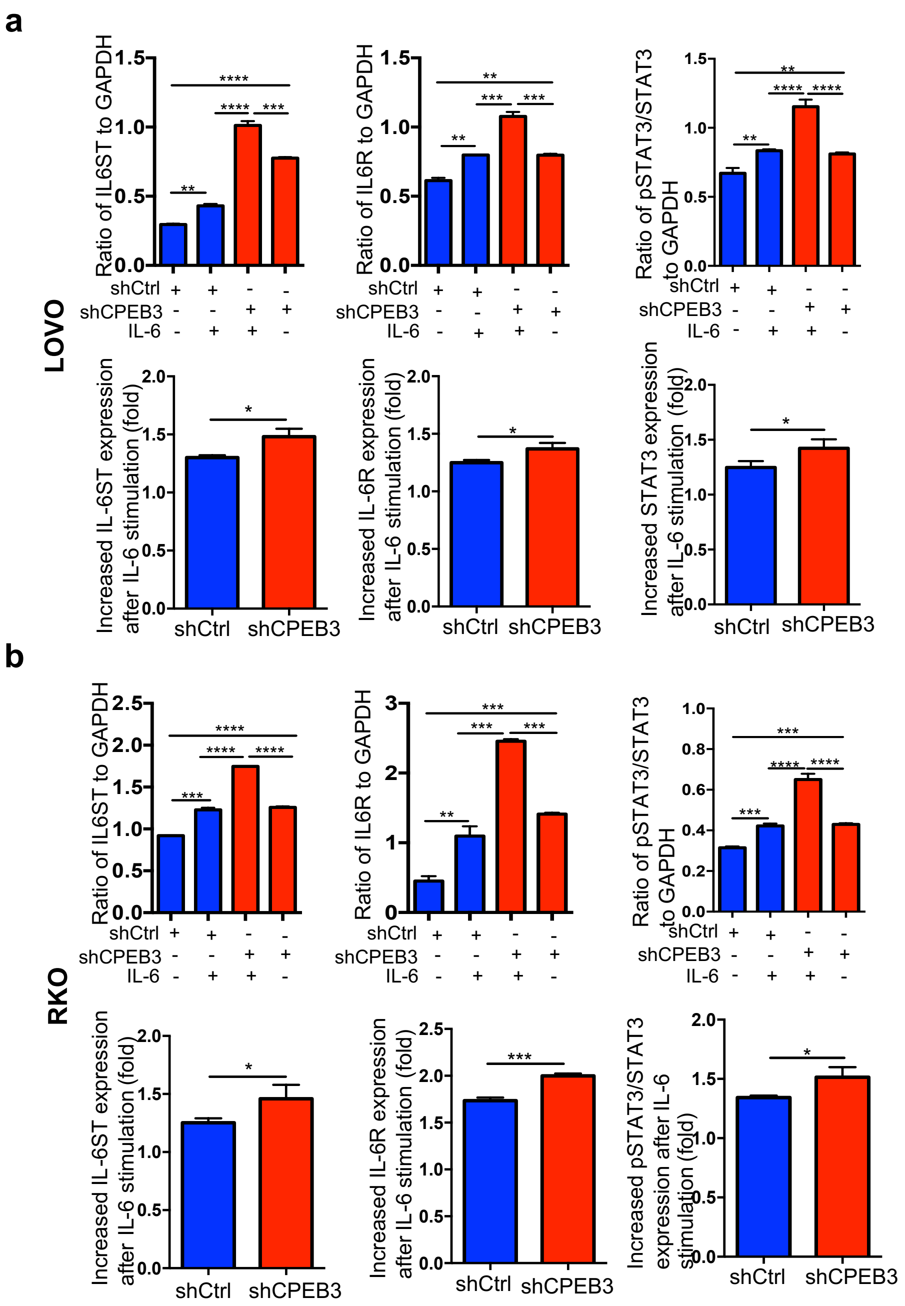

Supplementary Figure S7

The average gray values analysis of protein (a) The average gray values of IL-6ST, IL-6R and pSTAT3/STAT3 in LoVo-shCtrl/shCPEB3 cells were analyzed. The ratio of increased protein expression was analyzed (as indicated in Fig. 4c); error bars, SEM. (b) The average gray values of IL-6ST, IL-6R and pSTAT3/STAT3 in RKO-shCtrl/shCPEB3 cells were analyzed. The ratio of increased protein expression was analyzed (as indicated in Fig. 4c); error bars, SEM. *, P < 0.05; **, P < 0.01; ***, P < 0.001; ****, P < 0.0001.

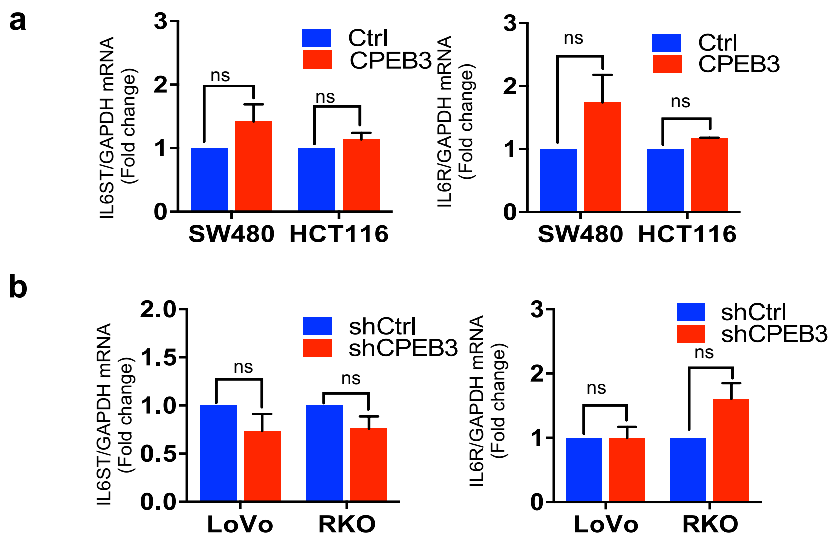

Supplementary Figure S8

The mRNA analysis of IL-6ST and IL-6R (a) The expression of IL-6R and IL-6ST was tested in the overexpressed CPEB3 SW480 and HCT116 cells using qRT-PCR; error bars, SEM. (b) The expression of IL-6R and IL-6ST was tested in the knockdown CPEB3 LoVo and RKO cells using qRT-PCR; error bars, SEM.

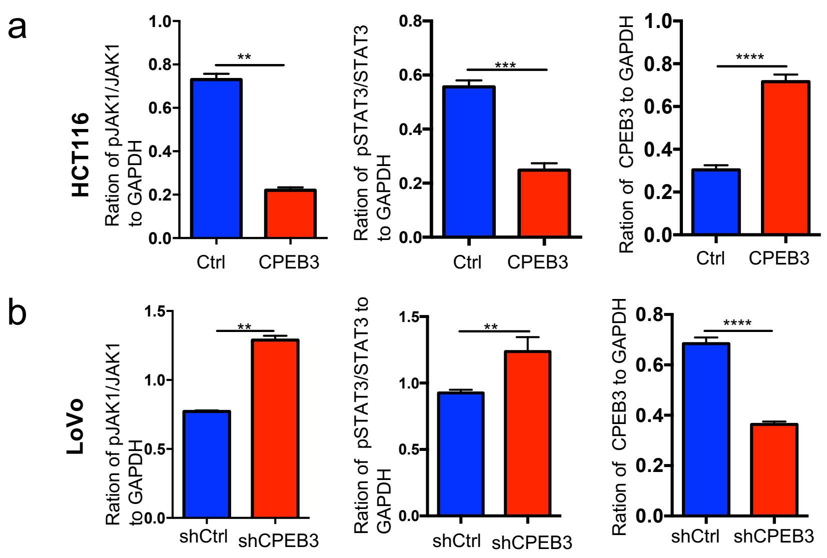

Supplementary Figure S9

The average gray values analysis of protein in tumor burdened nude mice (a) The average gray values of pJAK1/JAK1, pSTAT3/STAT3 and CPEB3 were analyzed in a subcutaneous xenograft model of HCT116-CPEB3 transduced CRC cells in BALB/c nude mice tumor (as indicated in Fig. 6f); error bars, SEM. (b) The average gray values of pJAK1/JAK1, pSTAT3/STAT3 and CPEB3 were analyzed in a subcutaneous xenograft model of LoVo-shCPEB3 transduced CRC cells in BALB/c nude mice tumor (as indicated in Fig. 6f); error bars, SEM. *, $P < 0.05$; **, $P < 0.01$; ***, $P < 0.001$; ****, $P < 0.0001$.
